# Predictive Value of Total Metabolic Tumor Burden Prior to Treatment in NSCLC Patients Treated with Immune Checkpoint Inhibition

**DOI:** 10.3390/jcm12113725

**Published:** 2023-05-28

**Authors:** Ken Kudura, Nando Ritz, Arnoud J. Templeton, Tim Kutzker, Robert Foerster, Kwadwo Antwi, Michael C. Kreissl, Martin H. K. Hoffmann

**Affiliations:** 1 Department of Nuclear Medicine, Sankt Clara Hospital, 4058 Basel, Switzerland; 2 Department of Radiology, Sankt Clara Hospital, 4058 Basel, Switzerland; 3 Sankt Clara Research, 4002 Basel, Switzerland; 4 Division of Nuclear Medicine, Department of Radiology and Nuclear Medicine, University Hospital Magdeburg, 39120 Magdeburg, Germany; 5 Faculty of Medicine, University of Basel, 4001 Basel, Switzerland; 6 Faculty of Applied Statistics, Humboldt University, 10117 Berlin, Germany; 7 Department of Radiooncology, Cantonal Hospital Winterthur, 8400 Winterthur, Switzerland

**Keywords:** FDG-PET/CT, lung cancer, NSCLC, metabolic tumor burden, novel therapeutic approaches, predictive biomarker, immunotherapy

## Abstract

Objectives: We aimed to assess the predictive value of the total metabolic tumor burden prior to treatment in patients with advanced non-small-cell lung cancer (NSCLC) receiving immune checkpoint inhibitors (ICIs). Methods: Pre-treatment 2-deoxy-2-[^18^F]fluoro-D-glucose positron emission tomography/computed tomography (PET/CT) scans performed in two consecutive years for staging in adult patients with confirmed NSCLC were considered. Volume, maximum/mean standardized uptake value (SUVmax/SUVmean), metabolic tumor volume (MTV) and total lesion glycolysis (TLG) were assessed per delineated malignant lesion (including primary tumor, regional lymph nodes and distant metastases) in addition to the morphology of the primary tumor and clinical data. Total metabolic tumor burden was captured by _total_MTV and _total_TLG. Overall survival (OS), progression-free survival (PFS) and clinical benefit (CB) were used as endpoints for response to treatment. Results: A total of 125 NSCLC patients were included. Osseous metastases were the most frequent distant metastases (n = 17), followed by thoracal distant metastases (pulmonal = 14 and pleural = 13). Total metabolic tumor burden prior to treatment was significantly higher in patients treated with ICIs (mean _total_MTV ± standard deviation (SD) 72.2 ± 78.7; mean _total_TLG ± SD 462.2 ± 538.9) compared to those without ICI treatment (mean _total_MTV ± SD 58.1 ± 233.8; mean _total_TLG ± SD 290.0 ± 784.2). Among the patients who received ICIs, a solid morphology of the primary tumor on imaging prior to treatment was the strongest outcome predictor for OS (Hazard ratio HR 28.04, *p* < 0.01), PFS (HR 30.89, *p* < 0.01) and CB (parameter estimation PE 3.46, *p* < 0.01), followed by the metabolic features of the primary tumor. Interestingly, total metabolic tumor burden prior to immunotherapy showed a negligible impact on OS (*p* = 0.04) and PFS (*p* = 0.01) after treatment given the hazard ratios of 1.00, but also on CB (*p* = 0.01) given the PE < 0.01. Overall, biomarkers on pre-treatment PET/CT scans showed greater predictive power in patients receiving ICIs, compared to patients without ICI treatment. Conclusions: Morphological and metabolic properties of the primary tumors prior to treatment in advanced NSCLC patients treated with ICI showed great outcome prediction performances, as opposed to the pre-treatment total metabolic tumor burdens, captured by _total_MTV and _total_TLG, both with negligible impact on OS, PFS and CB. However, the outcome prediction performance of the total metabolic tumor burden might be influenced by the value itself (e.g., poorer prediction performance at very high or very low values of total metabolic tumor burden). Further studies including subgroup analysis with regards to different values of total metabolic tumor burden and their respective outcome prediction performances might be needed.

## 1. Introduction

According to the world health organization (WHO), lung cancer was the leading cause of death, at 1.80 million people worldwide, in 2020. Cancer-related mortality with non-small-cell lung cancer (NSCLC) accounted for 80–85% of the cases [1,2,3,4,5]. In the majority of NSCLC patients, either locally advanced or metastatic disease is captured at diagnosis [6]. The introduction of immune checkpoint inhibitors (ICIs) targeting programmed cell death 1 (PD-1) or its ligand (PD-L1) has been a groundbreaking treatment approach for the management of advanced NSCLC [7,8,9,10,11]. However, given their limited response rates and high immunotoxicities, there has been a rising interest in identifying biomarkers for an accurate selection of NSCLC patients who would benefit from ICI treatment [7,12,13,14].

The 2-deoxy-2-[^18^F]fluoro-D-glucose positron emission tomography/computed tomography (FDG-PET/CT) is a non-invasive hybrid imaging modality, which captures metabolic and morphological features of a tumor in the same modality [15]. FDG-PET/CT has been an integral part of staging, detection of additional primary tumors at staging and treatment response assessment in NSCLC patients [16,17]. Metabolic parameters on FDG-PET/CT, such as maximum/mean standardized uptake value (SUVmax/SUVmean), metabolic tumor volume (MTV) or total lesion glycolysis (TLG) have been the subjects of recent, innovative and partly contradictory investigations regarding their abilities to predict patient response, but also new patterns of response to ICIs, such as pseudo- or hyper-progression, have been observed [18,19,20,21,22,23,24,25,26,27,28,29,30,31,32,33,34,35,36,37,38,39,40,41].

Recently published investigations suggested that morphological and metabolic features of primary tumors were strong predictive biomarkers for response to an ICI linked with clinical data in NSCLC patients [27]. However, recent literature also highlighted the relevance of the tumor environment and metastases features in addition to the primary tumor properties with regards to the patient immune response to treatment. In fact, larger tumors might be more immunosuppressive, affecting the immune responses initiated by ICI [12,22,25].

Therefore, we aimed to assess the predictive value of the total metabolic tumor burden prior to treatment in patients with advanced NSCLC receiving ICIs.

## 2. Materials and Methods

### 2.1. Patient Selection

The following inclusion and exclusion criteria were defined for patient selection at the start of the study.

Inclusion:

- consecutive pre-treatment PET/CT scans performed at our institution between 1 January 2020 and 31 December 2021 for staging in adult patients
- pathologically confirmed NSCLC were considered, following patient consent.

Exclusion:

- patients under the age of 18 years
- no consent for the use of their data for research

For this single-center retrospective study, a discovery PET/64-detector CT scanner (Discovery Molecular Insights-(DMI) PET/CT, General Electrics (GE) Healthcare, Waukesha, Wisconsin, United States of America) was used for image acquisition from the skull to the thighs in supine position (static 3D PET acquisition in 150 s per bed position). An ordered subset expectation maximization (OSEM) was performed as a standard reconstruction algorithm for PET images with a threshold set at 42% of the maximum standardized uptake value (SUVmax) and time-of-flight correction. The department standard protocol required iodinated contrast medium in the absence of renal impairment or allergy. The diagnostic CT scan, with dedicated chest acquisition, was also used for attenuation correction.

Baseline characteristics, such as age (in years), sex (male/female), body mass index (BMI, in kilograms per square meter kg/m^2^), subtype of NSCLC (adenocarcinoma, squamous cell carcinoma, large cell carcinoma and neuroendocrine tumor), clinical stage according to the 8th edition of the American Joint Committee on Cancer (AJCC) and treatment regimen (first-line ICI or second-ICI vs. no ICI) were extracted from medical reports.

### 2.2. Lesion Segmentation

Any lesion reported as malignant (for instance, primary tumor, regional lymph node metastasis and distant metastasis) by the reporting physicians in clinical routine was retrospectively manually delineated on the co-registered CT and PET images at an advanced workstation (AW), GE Healthcare AW 4.7. For this purpose, a manual 3D-contouring tool was used to contour malignant lesions. The contouring could be manually corrected by matching the lesion borders on the CT and PET images. The morphological features of primary tumors were reported (solid, subsolid, mixed solid/subsolid or cystic) by two physicians (both certified in radiology and nuclear medicine). Furthermore, volume, SUVmax, SUVmean, MTV and TLG per delineated lesion were extracted from the same volume of interest (VOI). Total metabolic tumor burden was captured by total metabolic tumor volume (_total_MTV) and total lesion glycolysis (_total_TLG). The _total_MTV was defined as the sum of MTV of all delineated lesions in the same patient and the _total_TLG as the sum of TLG of all delineated lesions in the same patient.

### 2.3. Response Assessment

Response to treatment was retrospectively assessed using three endpoints: Overall survival (OS) was defined as the time window from the date of diagnosis to death or the last follow up. Progression-free survival (PFS) was defined as the time window from the date of diagnosis to disease progression. Finally, no disease progression from treatment initiation to the last follow up (for instance complete response, partial response and stable disease) was considered as clinical benefit (CB). Response to treatment was assessed according to response evaluation criteria in solid tumors (iRECIST). All three endpoints were assessed on the same date, 19 August 2022.

### 2.4. Statistical Analysis

All statistical analyses were performed using R (version 4.1.1). Categorical variables were characterized using frequencies. Mean, standard deviation (SD) and interquantile range (Q1–Q3) were used to describe continuous variables. A Mann–Whitney U test was then performed to compare continuous variables between patients treated with immunotherapy versus no immunotherapy. A multivariate backward stepwise logistic regression approach was chosen to assess the outcome prediction power of metabolic tumor burden using OS, PFS and CB as endpoints. Subsequently, a cox proportional hazards regression model was used to assess the effect of significant variables on OS and PFS (hazard ratio), while a logistic regression model was chosen for significant variables with regards to clinical benefit. Finally, receiver operating characteristic (ROC) curves were generated to illustrate the predictive power of the generated prediction models. Statistical significance was accepted at *p* < 0.05.

## 3. Results

The 125 pre-treatment PET/CT scans from 125 different patients were considered for the purpose of our investigations following our inclusion criteria. The included PET/CT scans were then dichotomized into two groups, according to whether the corresponding patient was treated with ICI (n = 50, in first-line n = 25 or second-line n = 25) or not (n = 75). Table 1.

**Figure 1:**
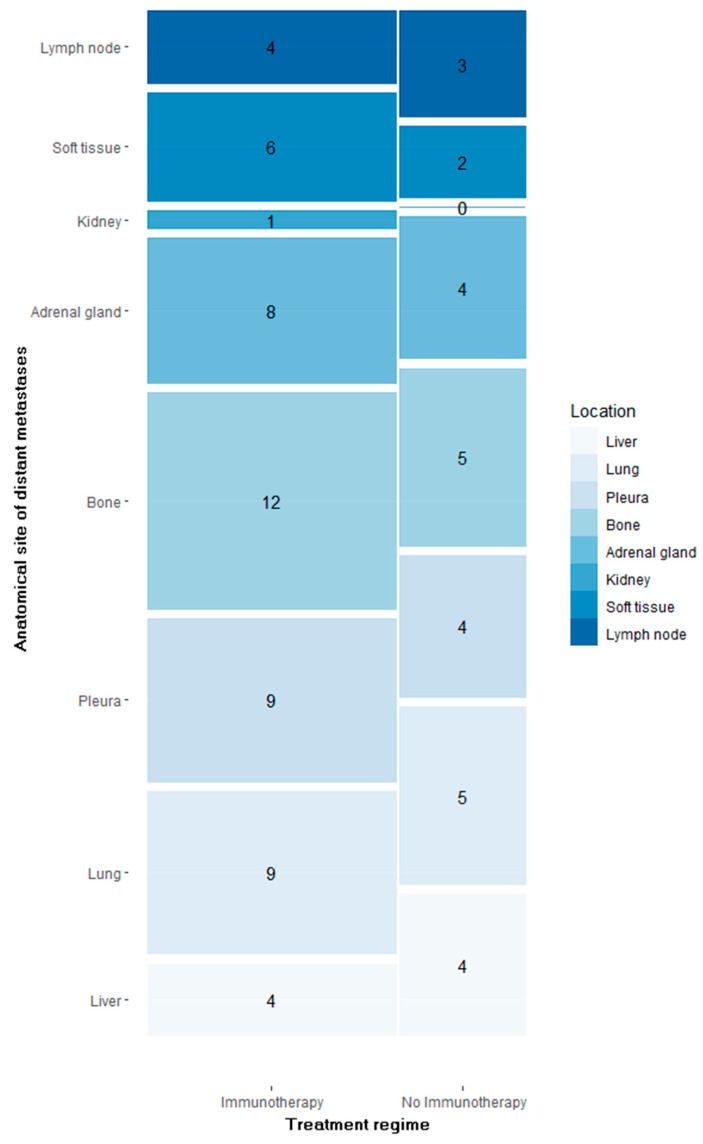
Number of segmented distant metastases per anatomical site in patients treated with immunotherapy vs. no immunotherapy.

Osseous metastases were by far the most frequent distant metastases (n = 17), followed by thoracal distant metastases (pulmonal = 14 and pleural = 13), as much as adrenal (n = 12) and hepatic (n = 8) metastases, as illustrated in Figure 1.

### 3.1. Lesion Segmentation

Significant differences were found with regards to total metabolic tumor burden before treatment initiation within the dichotomized cohort. In fact, total metabolic tumor burden prior to treatment was significantly higher in patients treated with ICIs, as illustrated in Table 2.

### 3.2. Predictive Value of Total Metabolic Tumor Burden for Treatment Response

The chosen multivariate backward stepwise logistic regression analyses, as a systematic approach, required prior minimization of the so-called “perfect correlation effect” in our data. For this purpose, an empiric correlation matrix capturing the entire data set was generated.

Pretreatment total metabolic tumor burden was captured by _total_MTV and _total_TLG prior to any treatment, as previously defined in the methods section. However, the generated correlation matrix displayed a correlation factor of 0.98 between _total_MTV and _total_TLG, as a result of perfectly linear behavior between these two parameters. Therefore, _total_TLG was chosen over _total_MTV in the regression analyses to assess total metabolic tumor burden prior to any treatment, in order to avoid any overfitting of our data. All other metabolic variables, in addition to all morphological parameters and all listed baseline characteristics, were taken into consideration for the multivariate backward stepwise logistical regression.

#### 3.2.1. Predictive Biomarkers for Overall Survival and Progression-Free Survival

Several biomarkers at baseline were found to be predictive of treatment response in NSCLC patients receiving immunotherapy, as reported in Table 3. In fact, among these patients, a solid morphology of the primary tumor on imaging prior to treatment was the strongest outcome predictor (Hazard ratio HR 28.04, *p* < 0.01), followed by the metabolic features of the primary tumor. Interestingly, total metabolic tumor burden prior to immunotherapy showed a negligible impact on OS (*p* = 0.04) and PFS (*p* = 0.01) after treatment, given the hazard ratios of 1.00.

The anatomical localization of distant metastases played a significant predictive role in patients who did not receive immunotherapy for treatment. In this patient group, the total metabolic tumor burden in soft tissues prior to treatment was captured as the strongest outcome predictor for OS (HR 2.05, *p* = 0.02) and PFS (HR 1.91, *p* = 0.02), while the total metabolic tumor burden at baseline, regardless of any anatomical consideration for distant metastases, displayed a negligible influence on OS (HR 1.00, *p* < 0.01) and PFS (HR 1.00, *p* < 0.01) after treatment. In contrast to patients treated with immunotherapy, neither morphological nor metabolic features of the primary tumors influenced the outcome in the patient group without immunotherapy.

#### 3.2.2. Predictive Biomarkers for Clinical Benefit

Various biomarkers at baseline were found to be predictive of clinical benefit, as reported in Table 4. A solid morphology of the primary tumor prior to treatment was the strongest predictive biomarker for clinical benefit among patients treated with immunotherapy (*p* < 0.01, parameter estimation (PE) = 3.46), while no predictive power could be demonstrated in patients who did not receive immunotherapy (*p* = 0.23, PE = 0.87). Metabolic parameters of primary tumors at baseline were also predictive of clinical benefit in both patient groups; however, they had a greater predictive power in patients treated with immunotherapy.

Interestingly, total metabolic tumor burden prior to treatment also displayed a negligible impact on clinical benefit in patients treated with ICI (*p* = 0.01, PE < 0.01), as much as on patients with no immunotherapy (*p* = 0.01, PE < 0.01). Furthermore, the anatomical site of metastases did not show any impact on clinical benefits in both groups.

**Figure 2:**
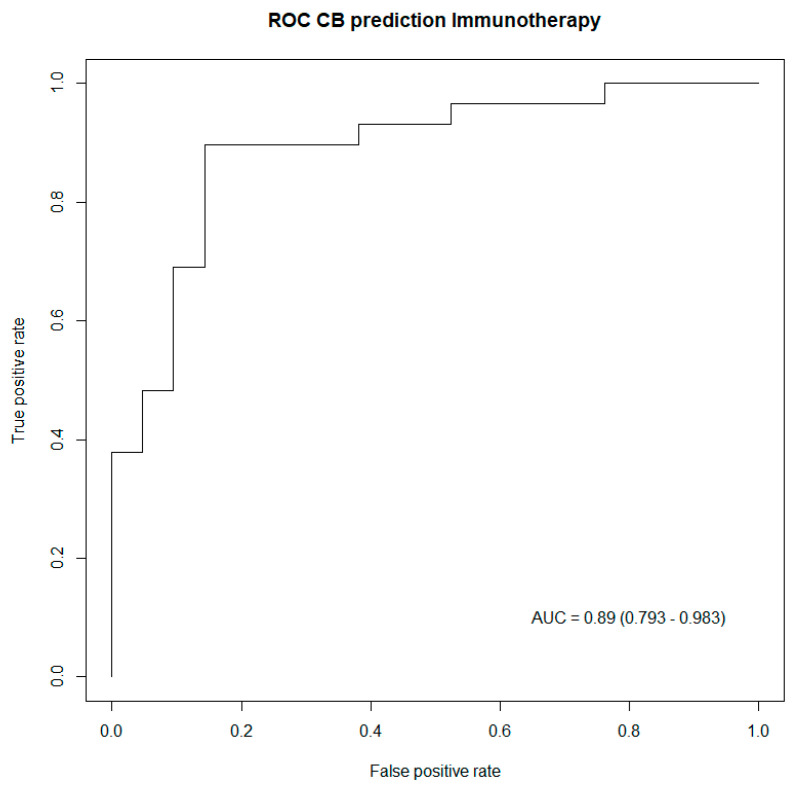
ROC curve capturing the performance of the generated model including biomarkers predictive of clinical benefit in patients treated with immunotherapy. AUC: Area under the curve (95% confidence interval).

**Figure 3:**
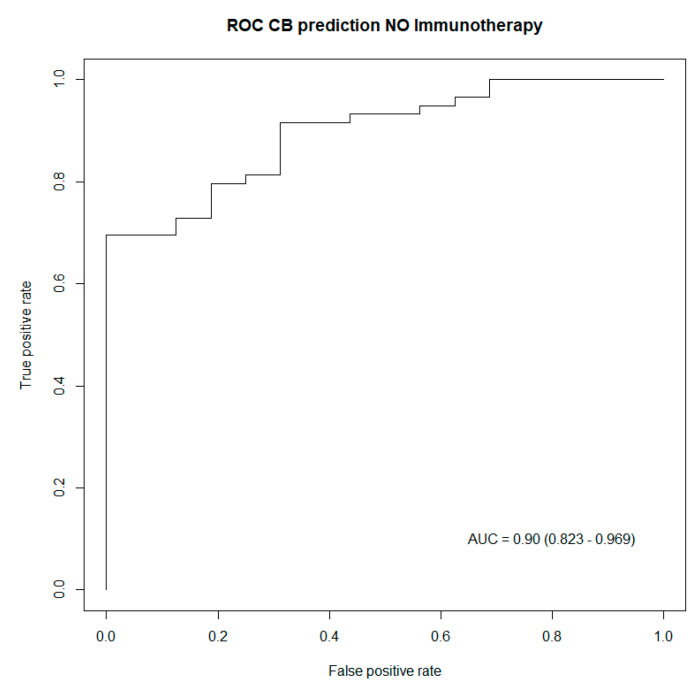
ROC curve capturing the performance of the generated model including biomarkers predictive of clinical benefit in patients treated with no immunotherapy. AUC: Area under the curve (95% confidence interval).

In order to validate these results, a concordance test, as well as an LR test, were performed to assess the performance of these two prediction models. Both tests indicated better performances of the generated models for patients treated with immunotherapy, as captured in Table 4. Subsequently, ROC curves were also drawn up to illustrate the predictive power of the generated prediction models, including all predictive biomarkers for clinical benefit listed in Table 4. Both ROC curves captured excellent performances from both prediction models, respectively, in patients treated with immunotherapy, as well as in patients having no immunotherapy treatment Figure 2 and Figure 3.

## 4. Discussion

The identification of potential biomarkers able to predict a benefit from immunotherapy in advanced NSCLC patients may be crucial, given the immunotoxicities and moderate response rates of ICIs [42].

Therefore, we aimed at assessing the predictive value of the total metabolic tumor burden prior to treatment in patients with advanced NSCLC receiving ICIs.

Pre-treatment PET/CT scans performed in two consecutive years for staging in adult patients with confirmed NSCLC were considered. SUVmax, SUVmean, MTV and TLG were assessed per delineated malignant lesion (including primary tumor, regional lymph node metastases and distant metastases). Total metabolic tumor burden was defined by _total_MTV and _total_TLG. Response to treatment was captured by OS, PFS and CB.

Very interesting insights could be highlighted. Total metabolic tumor burden prior to treatment was significantly higher in patients treated with ICIs compared to those without ICI treatment, which could be explained by the composition of the patient groups. In fact, patients treated with ICIs displayed either a locally advanced or metastatic disease at diagnosis, while almost half of the cohort without ICI treatment presented a limited disease at diagnosis. Among the patients who received ICIs, a solid morphology of the primary tumor on imaging prior to treatment had the strongest outcome predictor for OS, PFS and CB, followed by metabolic features of the primary tumor. Interestingly, total metabolic tumor burden prior to immunotherapy showed a negligible impact on OS, PFS and CB. Overall, biomarkers on pre-treatment PET/CT scans showed greater predictive power in patients receiving ICI, compared to patients without ICI treatment, in whom the anatomical site of distant metastases played a significant predictive role on OS and PFS. These results are quite promising, especially since a recent analysis of entropy did not prove to be predictive of outcome [43].

In knowledge of recently published investigations from 2019 to this date, the following observations should be further discussed before confronting our results with the current literature [18,20,21,22,23,24,25,26,27,28,29,30,31,32,33,34,35,36].

First of all, while our ICI cohort size and observation time were in line with numerous published investigations on the topic, a certain heterogeneity in the composition of the examined populations could be noticed and considered as a first limitation for in-depth comparison with the current literature. In fact, some authors considered PD-1 inhibitors only, others PD-1 and PD-L1 inhibitors, some studies included ICI without additional chemotherapy, others ICI with additional chemotherapy. ICIs were given either as first-line treatment or as second-line treatment. An innovative approach in our study design might be the dichotomy of the initial cohort (PD-1 inhibitors or PD-L1 inhibitors as first- or second-line treatment vs. without any ICI as therapy in native patients with pathologically confirmed NSCLC), which allowed an interesting comparison of the predictive power of biomarkers on pre-treatment FDG-PET/CT scans between both patient groups.

Subsequently, a large methodological variability is noticeable in recently published investigations with regards to data assessment (for instance, manually vs. semi-automatically, definition of total metabolic tumor burden) or examined data (for instance, metabolic parameters only vs. addition of further parameters), which might be a second limitation for in-depth comparison with the current literature. Nevertheless, a potential methodological strength of our investigations might not only be the inclusion of clinical data and morphological features of primary tumors in all analyses, but also the stratification of the total metabolic tumor burden with regards to the primary tumor, regional lymph node metastases, and distant metastases, as well as the anatomical sites of distant metastases.

Despite the limited, but in-depth, comparison with recent literature [18,20,21,22,23,24,25,26,27,28,29,30,31,32,33,34,35,36], our statistical analyses showed an innovative, and, above all, consistent and univocal trend with strong statistical significance to be further discussed. Morphological and metabolic properties of the primary tumors prior to treatment in patients with advanced NSCLC treated with ICIs showed great outcome prediction power, as opposed to the pre-treatment total metabolic tumor burden, captured by _total_MTV and _total_TLG, both of which had negligible impacts on OS, PFS and CB. These insights are very innovative in light of recently published investigations. In fact, while TLG [12,13,23,26,28,34] and SUV [12,26,32,33,34] were mostly not associated with either OS or PFS, MTV has been often reported as a potential predictor of response to ICIs in NSCLC patients [12,18,20,21,22,23,24,26,30,32,33,34,36].

In our data, _total_MTV and _total_TLG displayed a correlation factor of 0.98, as a result of a perfectly linear behavior. Thus, _total_TLG properties also applied to _total_MTV, both capturing total metabolic tumor burden. Zhu et al. reported, in their meta-analysis, published in 2022, different performances of MTV as an outcome predictor in NSCLC patients depending on different cut-off values. The authors noticed the worst predictive power of MTV when the cut-off value was set over 100 cm^3^. The best predictive power was when the cut-off value was set between 50–100 cm^3^, followed by a range below 50 cm^3^ [12]. Thus, we compared our median _total_MTV with the median total metabolic tumor burden in the cited studies, reporting MTV as a strong outcome predictor. We noticed higher median total metabolic tumor burdens, compared to our data, in studies reporting MTV as predictive of response to ICIs [12]. In summary, our results also might suggest that the predictive power of the total metabolic tumor burden prior to ICIs might be influenced by the value of the tumor burden itself, with poorer prediction performance at very high or very low values of the metabolic tumor burden in patients with advanced NSCLC. This is very much in keeping with the results recently published by Silva et al. [44]. However, this study had a fairly heterogeneous patient collective.

Unfortunately, the cohort size of patients treated with ICIs in our data did not allow further statistically meaningful subgroup analyses with regards to different values of metabolic tumor burden and their respective outcome prediction performances.

In order to overcome the mentioned demographic and methodological limitations, and so to validate our results, further prospective studies with larger, homogenous cohorts of advanced NSCLC patients are needed with a standardized algorithm for data assessment, including subgroup analysis, with regards to different values of the total metabolic tumor burden and their respective outcome prediction performances.

## 5. Conclusions

Morphological and metabolic properties of the primary tumors prior to treatment in advanced NSCLC patients treated with ICIs showed great outcome prediction performances, as opposed to the pre-treatment total metabolic tumor burden, captured by _total_MTV and _total_TLG, both of which had negligible impacts on OS, PFS and CB. However, the outcome prediction performance of the total metabolic tumor burden might be influenced by the value itself (e.g., poorer prediction performance at very high or very low values). Further studies, including subgroup analysis, with regards to different values of total metabolic tumor burden and their respective predictive powers might be needed.

## Figures and Tables

**Table 1:** displays baseline characteristics of the considered patient cohort (n = 125).

	Immunotherapy	No Immunotherapy
**Age mean in years (SD)**	72.0 (9.39)	72.8 (9.70)
**Q1–Q3**	65.25–79.75	65.5–81.0
**Gender**		
Male	30 (60.0%)	34 (45.3%)
Female	20 (40.0%)	41 (54.7%)
**BMI mean in kg/m^2^ (SD)**	25.9 (5.06)	26.3 (6.42)
**Q1–Q3**	22.4–29.0	22.3–28.8
**Histopathological subtype**		
Adenocarcinoma	22 (44.0%)	37 (49.3%)
Squamous cell carcinoma	11 (22.0%)	20 (26.7%)
Large cell carcinoma	10 (20.0%)	7 (9.3%)
Neuroendocrine tumor	6 (12.0%)	9 (12.0%)
Not specific	1 (2.0%)	2 (2.7%)
**Clinical staging**		
I	0 (0.0%)	29 (38.7%)
II	0 (0.0%)	7 (9.3%)
III	21 (42.0%)	27 (36.0%)
IV	29 (58.0%)	12 (16.0)

**Table 2:** The metabolic parameters of all delineated primary tumors, regional lymph node metastases, and distant metastases, as much as the total metabolic tumor burden prior to treatment in patients treated with immunotherapy vs. no immunotherapy.

	Immunotherapy	No Immunotherapy	*p*-Value
**Metabolic parameters of primary tumor** SUVmax mean (SD, Q1–Q3) SUVmean mean (SD, Q1–Q3) MTV mean (SD, Q1–Q3) TLG mean (SD, Q1–Q3)	50 12.5 (5.0, 8.9–16.0) 7.3 (2.9, 5.0–9.2) 31.0 (32.6, 5.3–45.4) 220.8 (226.4, 40.5–315.9)	75 9.5 (6.4, 4.0–15.1) 5.6 (3.7, 2.3–8.4) 21.3 (28.9, 2.2–25.9) 154.1 (258.9, 6.2–168.9)	<0.01 <0.01 0.09 0.13
**Total regional lymph node metastases** MTV mean (SD, Q1–Q3) TLG mean (SD, Q1–Q3)	44 20.6 (27.9, 5.0–23.2) 103.6 (151.1, 11.0–122.4)	35 15.7 (22.6, 3.4–18.1) 81.0 (187.4, 7.2–76.6)	<0.01
**Total distant metastases** MTV mean (SD, Q1–Q3) TLG mean (SD, Q1–Q3)	31 37.2 (66.7, 4.1–37.1) 242.2 (571.4, 11.3–221.6)	15 147.5 (511.9, 2.4–20.1) 490.7 (1602.2, 4.8–85.5)	<0.01
**Total metabolic tumor burden** MTV mean (SD, Q1–Q3) TLG mean (SD, Q1–Q3)	50 72.2 (78.7, 19.3–79.7) 462.2 (538.9, 145.7–581.6)	75 58.1 (233.8, 2.3–46.3) 290.0 (784.2, 7.8–273.4)	<0.01

**Table 3:** All predictive biomarkers for response to treatment (immunotherapy vs. no immunotherapy) and their impact on overall survival OS and progression-free survival PFS; PT: Primary tumor.

	Immunotherapy				No Immunotherapy			
	OS		PFS		OS		PFS	
	HR	*p*-Value	HR	*p*-Value	HR	*p*-Value	HR	*p*-Value
**Age**	0.94	0.02	0.94	0.03	1.02	0.46	1.03	0.27
**SUVmaxPT**	1.43	<0.01	1.33	<0.01	1.10	0.17	1.13	0.06
**VolumePT**	1.08	<0.01	1.06	<0.01	1.04	0.14	1.04	0.10
**TLGPT**	0.99	<0.01	1.00	0.03	0.99	0.28	0.99	0.15
**Solid morphology PT**	28.04	<0.01	30.89	<0.01	2.05	0.44	1.42	0.68
**_total_** **TLG**	1.00	0.04	1.00	0.01	1.00	<0.01	1.00	<0.01
**_soft tissue_** **TLG**	0.98	0.73	0.98	0.68	2.05	0.02	1.91	0.02
**_lymph nodes_** **TLG**	1.00	0.97	1.00	0.82	1.08	0.28	1.13	0.13

**Table 4:** All predictive biomarkers for clinical benefit in patients treated with immunotherapy vs. no immunotherapy. LR Test: Likelihood-ratio test; PE: Parameter estimation summarizes the effect of each predictor on clinical benefit.

	Immunotherapy			No Immunotherapy		
	*p*-Value		PE	*p*-Value		PE
**Age**	**0.02**		0.06	0.17		0.04
**SUVmaxPT**	0.01		0.28	0.03		0.14
**VolumePT**	0.01		0.06	0.02		0.06
**TLGPT**	0.02		0.01	0.05		0.01
**Solid morpho-logy PT**	<0.01		3.46	0.23		0.87
**_total_** **TLG**	0.01		<0.01	0.01		<0.01
**_lymph nodes_** **TLG**	0.76		<0.01	0.11		0.12
**Concordance Test**		**0.80**			**0.74**	
**LR Test**		<0.01			0.04	

## Data Availability

The data presented in this study are available on request from the corresponding author.

## Abbreviations

WHO	World Health Organization
NSCLC	Non-small cell lung cancer
ICI	Immune checkpoint inhibitors
PD-1	Programmed cell death receptor-1
PD-L1	Programmed cell death ligand-1
FDG-PET/CT	2-deoxy-2-[^18^F]fluoro-D-glucose positron emission tomography/computed tomography
SUVmax	Maximum standardized uptake value
SUVmean	Mean standardized uptake value
MTV	Metabolic tumor volume
TLG	Total lesion glycolysis
DMI	Discovery Molecular Insights
GE	General Electrics
OSEM	Ordered subset expectation maximization
BMI	Body mass index
AJCC	American Joint Committee on Cancer
AW	Advanced workstation
VOI	Volume of interest
OS	Overall survival
PFS	Progression-free survival
CB	Clinical benefit
SD	Standard deviation
IQR	Interquantile range
ROC	Receiver operating characteristic
HR	Hazard ratio
PT	Primary tumor
LR	Likelihood-ratio
PE	Parameter estimation
AUC	Area under the curve
